# A Smartphone-Based Biosensor for Non-Invasive Monitoring of Total Hemoglobin Concentration in Humans with High Accuracy

**DOI:** 10.3390/bios12100781

**Published:** 2022-09-21

**Authors:** Zhipeng Fan, Yong Zhou, Haoyu Zhai, Qi Wang, Honghui He

**Affiliations:** 1 Guangdong Research Center of Polarization Imaging and Measurement Engineering Technology, Shenzhen Key Laboratory for Minimal Invasive Medical Technologies, Institute of Biopharmaceutical and Health Engineering, Tsinghua Shenzhen International Graduate School, Tsinghua University, Shenzhen 518055, China; 2 Shenzhen Maidu Technology Co., Ltd., Shenzhen 518000, China

**Keywords:** biosensor, smartphone, noninvasive, multi-wavelength, L*a*b* color space, total hemoglobin concentration

## Abstract

In this paper, we propose a smartphone-based biosensor for detecting human total hemoglobin concentration in vivo with high accuracy. Compared to the existing biosensors used to measure hemoglobin concentration, the smartphone-based sensor utilizes the camera, memory, and computing power of the phone. Thus, the cost is largely reduced. Compared to existing smartphone-based sensors, we developed a highly integrated multi-wavelength LED module and a specially designed phone fixture to reduce spatial errors and motion artifacts, respectively. In addition, we embedded a new algorithm into our smartphone-based sensor to improve the measurement accuracy; an L*a*b* color space transformation and the “a” parameter were used to perform the final quantification. We collected 24 blood samples from normal and anemic populations. The adjusted *R*^2^ of the prediction results obtained from the multiple linear regression method reached 0.880, and the *RMSE* reached 9.04, which met the accuracy requirements of non-invasive detection of hemoglobin concentration.

## 1. Introduction

Globally, more than one-third of pregnant women aged 15–49 suffer from anemia [1]. Anemia in pregnant women during pregnancy can cause complications, which can lead to death in severe cases. At the same time, the disease cycle of anemia is long, and it is not easy to treat or recover from; hence, it needs to be monitored and tested frequently. At present, the clinical solution in hospitals is mainly based on collecting blood sample from veins and then using the ferric cyanide method to measure the hemoglobin concentration [2]. Although this detection method has high measurement accuracy, it also has its limitations. The method requires the collection of human blood samples, during which both patients and medical staff are at risk of infection from exposure to blood and needles. In addition, professional medical personnel and equipment are required to perform the detection operation whose process is cumbersome and has low efficiency. Therefore, the method is inconvenient for the detection of hemoglobin concentration in daily life.

Currently, there are several pieces of non-invasive equipment on the market for daily hemoglobin concentration testing [3,4]. However, the measurement accuracy of these devices can hardly reach medical standards [5,6,7], and they are often not very portable. Compared with these medical testing devices, smartphones show irreplaceable portability and convenience. It is predicted that by the year of 2023, there will be more than four billion smartphone users globally, making smartphones play a potentially huge role in medical testing in non-invasive optical measurements. Recently, the application of smartphones in human physiological parameters measurement and monitoring has become increasingly common, such as smartphone-based detection platforms for molecular diagnostics [8], clinical diagnosis of albumin-related diseases [9,10], heart rate detection [11,12,13], blood oxygen saturation detection [14,15,16], respiratory rate detection [17,18], and anemia detection [19,20,21]. The advantages of using smartphones for detection are as follows: there is no need to carry other hardware devices, and the powerful CPU and memory of the smartphone provide a good hardware platform for the calculation and analysis of physiological parameters.

Here, we propose an accurate smartphone-based biosensor for detecting human total hemoglobin concentration. This sensor utilizes the camera, memory, and computing power of the smartphone. We also develop a highly integrated multi-wavelength LED module and a specially designed phone fixture to reduce spatial errors and motion artifacts, respectively. In addition, we embed a new algorithm into the smartphone-based sensor to improve the measurement accuracy; an L*a*b* color space transformation is used, and the “a” parameter is proposed to perform the final quantification. A total of 24 blood samples are collected from normal and anemic populations. The results show that the hardware and algorithm proposed in this study can meet the accuracy requirements of non-invasive detection of hemoglobin concentration in vivo.

## 2. Materials and Methods

### 2.1. Modified Beer–Lambert Law and Multiwavelength Selection

**Figure 1:**
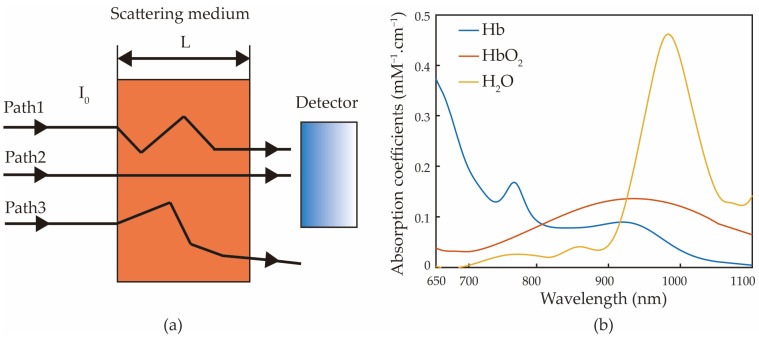
(**a**) Schematic of scattering, absorption of light in tissue, and detection. (**b**) Absorption coefficients of oxyhemoglobin, deoxy-hemoglobin, and water at 650–1100 nm.

The Beer–Lambert law [22] indicates that when a beam of light passes through a medium, the intensity of the detected light is proportional to the concentration of the medium C and the propagation distance of the beam L. The radius of human blood vessels increases and decreases periodically with the beating of the heart, so the photoelectric volume diagram of the human body can be detected. However, this model is based on the hypothesis that only light is absorbed. This hypothesis is not applicable in a human body, where the attenuation of near-infrared light caused by scattering dominates relative to absorption (roughly 80% scattering vs. 20% absorption). In this case, the primary problem of using a photoelectric detector to receive the outgoing light is that the outgoing photons are not completely collected, as shown in Figure 1a. In addition, some of the light cannot reach the detector, so the actual optical attenuation is not accurately defined.

In addition, the multiple scattering of light in the tissue leads to an increase in the path L of light propagation, which also has a great influence on the measurement results. The modified Beer–Lambert law [23] takes into account the effect of scattering in the tissue, as shown in Equation (1),

(1) $$A_{\lambda }=(\varepsilon_{1_{\lambda }}\cdot c_{1}+\varepsilon_{2_{\lambda }}\cdot c_{2}+...\varepsilon_{n_{\lambda }}\cdot c_{n})\cdot d\cdot DPF+G.$$

where $A_{\lambda }$ is light attenuation (in natural logarithm), also known as optical density [24] directly related to the concentration of substances and the distance that light passes through; $\varepsilon_{n_{\lambda }}$ is the molar extinction coefficient of different substances; $c_{n}$ is the concentration of different substances; *DPF* is the differential path length factor [25,26], representing the proportion of the increase in the optical path length due to scattering; *d·DPF* represents the effective path length of light; and the factor *G* is the scattering factor, representing the effect of the nature and geometry of the tissue.

Blood absorbs different wavelengths of light differently. Figure 1b shows the absorption curves of oxyhemoglobin, anaerobic hemoglobin, and water [27,28,29]. In the near-infrared optical window of 600–1000 nm, oxyhemoglobin and anaerobic hemoglobin have an iso-absorption point at 810 nm [30]. Hence, researchers generally choose 810 nm as the base signal for non-invasive measurement of hemoglobin concentration. The absorption of water near 970 nm and 1050 nm dominates, and the influence of water can be removed by using the signal at these wavelengths.

### 2.2. Smartphone Measurement Device

Here, we used a light source containing 5 LEDs of different wavelengths to obtain the photoplethysmography (PPG) signals from a fingertip. The measurement wavelengths were 660 nm, 810 nm, 900 nm, 970 nm, and 1050 nm. We chose the iso-absorption point of oxyhemoglobin and deoxyhemoglobin at 810 nm. Based on the therapeutic window, 660 nm and 900 nm were added to differentiate oxyhemoglobin and deoxyhemoglobin [31,32]. Moreover, near 970 nm and 1050 nm, the absorption of water was obvious; thus, we added these two wavelengths to exclude the influence of water absorption.

**Figure 2:**
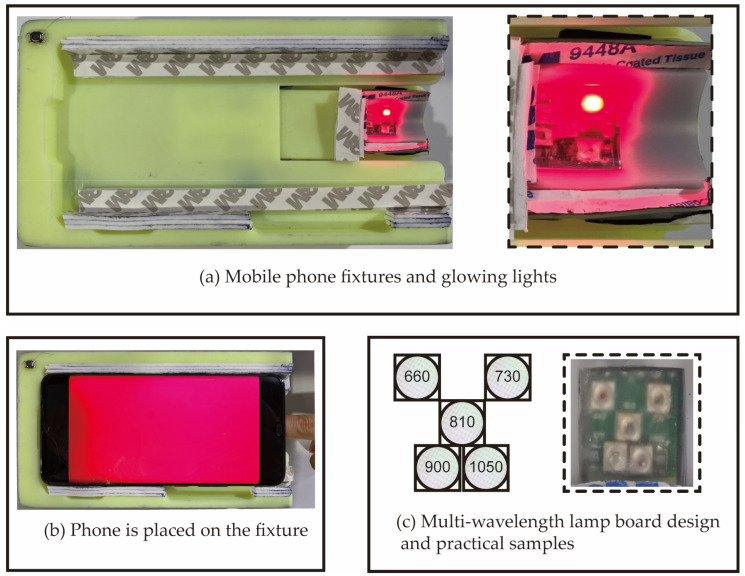
Hardware device for data collection: (**a**) Smartphone fixture with 660 nm LED on. (**b**) Smartphone on the fixture. (**c**) Arrangement of five-wavelength LEDs.

When a smartphone is held manually, the motion artifacts due to breathing and movement are prominent. Therefore, a 3D printed fix device is necessary, as shown in Figure 2a,b, which show the working smartphone system on the fixture. We also designed the arrangement of the lamps as shown in Figure 2c. As the absorption of the smartphone sensor at the wavelengths of 970 nm and 1050 nm is weaker than other wavelengths, we placed these two LEDs in the outermost positions of the fingertip, so the optical path was relatively short, and the signals were stronger. We designed the PCBA by ourselves and then handed it over to the factory to solder the patch to reduce the position error caused by human movement. The smartphone model (Huawei mate20pro, Shenzhen, China) used in this study has the acquisition pixel of 1920 × 1080. Its acquisition frame rate is 60 Hz, which is much larger than the PPG signal frequency of the human body (10 Hz). Hence, the sampling rate satisfies Shannon’s sampling law.

### 2.3. Data Collection

**Figure 3:**
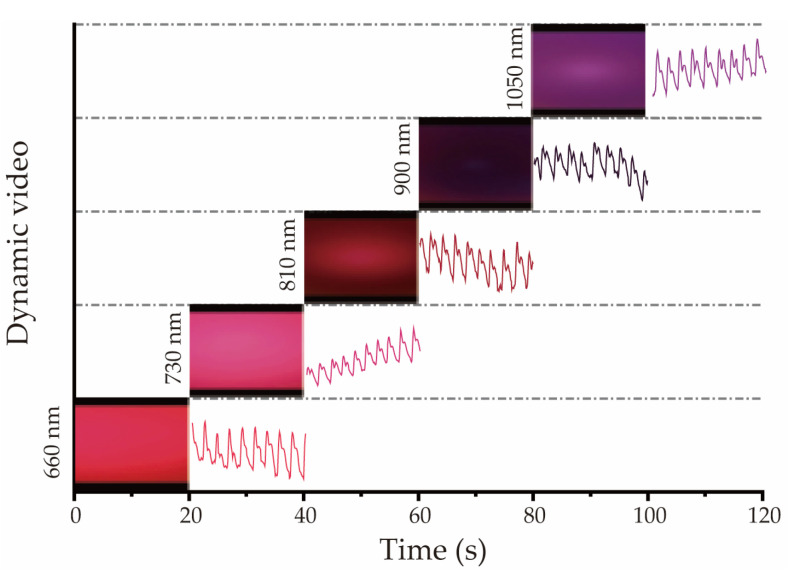
Schematic of the PPG data measurement and the waveforms display in sequence.

During the signal detection process, the volunteer’s finger remained motionless, and each LED was lit up to 20 s. As shown in Figure 3, in one cycle, all five LEDs were lit up separately. Thus, a video file with a total length of 100 s was collected.

Here we collected the PPG data on 12 normal adults (8 male volunteers, 4 female volunteers) and 12 hospitalized patients with anemia (6 male volunteers, 6 female volunteers). All the data were obtained from the volunteers’ right index fingers. The volunteers’ arms were placed on the table to ensure comfort and stability during the data acquisition, and a MATLAB-based program was used to check whether the frame rate was correct after the acquisition process. The volunteers’ heart rate, blood pressure, and age were also recorded.

### 2.4. Color Space Transformation

**Figure 4:**
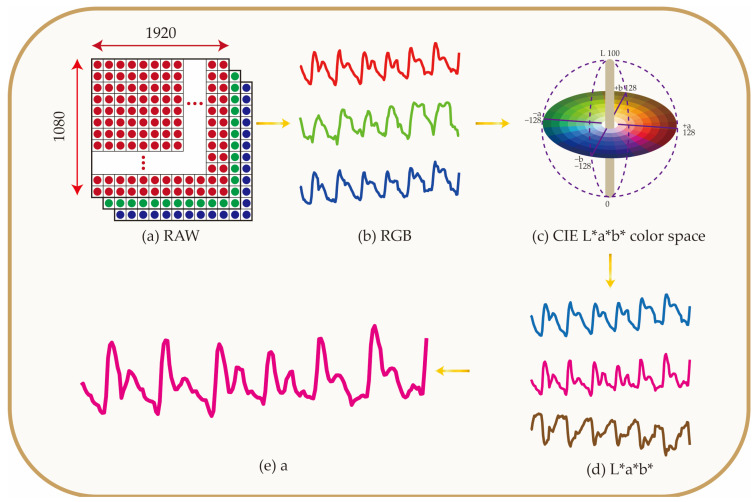
Smartphone collects the RGB channel information of the original image, converts it into the L*a*b* channel, and extracts the information of channel a for PPG calculations. (**a**) Schematic of a frame of image. (**b**) PPG signals of red, green, and blue channels. (**c**) CIE L*a*b* color space. (**d**) PPG signal of L*a*b* channels. (**e**) PPG signal of channel a.

The basic form of a time-continuous PPG video file is shown in Figure 4a. It is composed of color picture sequences of RGB channels. Here, the MATLAB program was used to extract images of each frame, and then an area of 1000 × 1000 pixels in the middle of the image was selected as the region of interest (ROI).

The RGB three-channel data of the ROI were extracted and averaged. The final obtained PPG signal is shown in Figure 4b. It can be seen that the signal noises of channels B and G are large, and the signals are unstable. However, the main information of the PPG signal image is concentrated in channel R, which is also more stable.

Previous studies have shown that the L*a*b* parameter provides a measurement of skin color perception [33] and, thus, mimics how skin is perceived by a dermatologist or the general population [34,35]. Chardon et al. proposed that in a three-dimensional L*a*b* space, all skin tones of light-skinned subjects are within a “banana”-shaped volume called skin tone volume. Skin redness (erythema reaction) can be represented as a displacement on the L*-a* plane. Since erythema is mainly caused by dilatation and congestion of local dermal capillaries of the skin, parameter “a” also reflects blood-related changes. CIE L*a*b* color space is shown in Figure 4c, where L* indicates light intensity related to the “luminous reflectance” (quantity of reflected light weighted with the spectral response of the human eye) and takes values from 0 (black) to 100 (white), a* indicates the color of the object on a scale that goes from green (−128) to red (128), and b* indicates the color of the object on a scale that goes from blue (−128) to yellow (128). The signals transformed from RGB to CIE L*a*b* color space by the RGB2Lab program are shown in Figure 4d, and the channel a signal was separately extracted as shown in Figure 4e. For all the volunteers’ data, the channel a signal was used as the initial signal of PPG calculation.

### 2.5. Data Analysis

**Figure 5:**
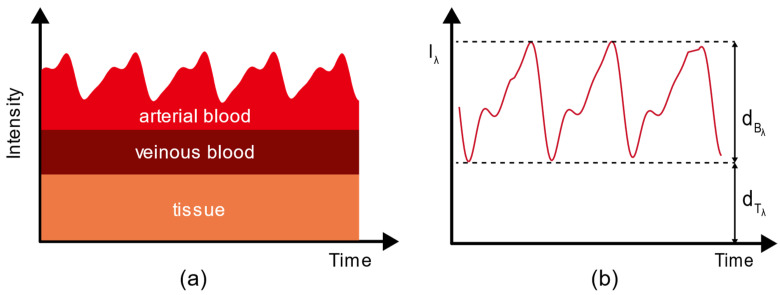
(**a**) PPG optical model of blood; (**b**) a characteristic PPG signal curve, where $d_{T_{\lambda }}$ represents the optical path of tissue in finger, and $d_{B_{\lambda }}$ represents the optical path of arterial blood in finger.

Figure 5a shows the PPG optical model of blood, which consists of tissue, venous blood, and arterial blood. Therefore, the model is simplified to only tissue and pulsating arterial blood, and then the modified Beer–Lambert law can be described as Equation (2),

(2) $$A_{\lambda }=\ln(\frac{I_{out}}{I_{in}})=(\varepsilon_{B_{\lambda }}\cdot c_{B}\cdot d_{B_{\lambda }}+\varepsilon_{T_{\lambda }}\cdot c_{T}\cdot d_{T_{\lambda }})\cdot DPF+G.$$

where $I_{out}$ represents the outgoing light intensity, $I_{in}$ represents the incident light intensity, $\varepsilon_{B_{\lambda }}$ represents the molar extinction coefficient of blood, $c_{B}$ represents the concentration of blood, $d_{B_{\lambda }}$ represents the light path of blood, $\varepsilon_{T_{\lambda }}$ represents the molar extinction coefficient of tissue, $c_{T}$ represents the concentration of tissue, $d_{T_{\lambda }}$ represents the light path of tissue.

The absorption coefficient of the five wavelengths of light used in our system is different in human tissues. Under the same incident light intensity, the emitting light intensity of the strong absorbed wavelength will be relatively small, sometimes even similar to the intensity of the background noise. Therefore, the signal-to-noise ratio of such wavelength is low, meaning that it is necessary to strengthen the incident intensity of such a wavelength of light to increase the signal-to-noise ratio, that is, to increase the input current of the corresponding LED. For light with weak absorption wavelength in the tissue, a stronger incident light will lead to a stronger emitted light, which then saturates the signal of that wavelength, so its incident light intensity should be reduced. In summary, in order to achieve a good signal-to-noise ratio of light for all five wavelengths, the incident light intensity of each wavelength must be appropriately adjusted. To eliminate the effect of the current adjustment on the result, we use the AC/DC of each wavelength to obtain the characteristic parameter of $R_{\lambda }$ as Equation (3),

(3) $$R_{\lambda }=AC_{\lambda }/DC_{\lambda }=\frac{I_{in}\cdot e^{\varepsilon_{B_{\lambda }}\cdot c_{B}.d_{B_{\lambda }}\cdot DPF+G}}{I_{in}\cdot e^{\varepsilon_{T_{\lambda }}\cdot c_{T}\cdot d_{T_{\lambda }}\cdot DPF+G}}=e^{(\varepsilon_{B_{\lambda }}\cdot c_{B}\cdot d_{B_{\lambda }}-\varepsilon_{T_{\lambda }}\cdot c_{T}\cdot d_{T_{\lambda }})\cdot DPF}.$$

To accurately obtain $R_{\lambda }$, we use a strict waveform screening tool and the skewness as a standard for waveform quality inspection. The waveforms that do not meet the requirements are not added to the calculation. Here, the parameter $R_{1050}$ performs multiple linear regression fitting.

## 3. Results

### 3.1. Comparison of RGB and L*a*b* Color Spaces Results

**Figure 6:**
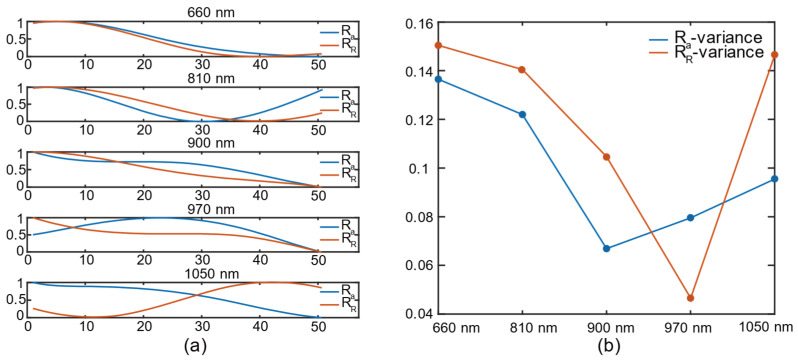
(**a**) Comparison of R_a_ and R_R_ at different wavelengths: 660 nm; 810 nm; 900 nm; 970 nm; 1050 nm; (**b**) variances in R_a_ and R_R_ at different wavelengths.

We selected 50 waveforms in this section to calculate the R color channel of the RGB color space and channel a of the L*a*b color space according to Equation (3). The values were normalized to eliminate the error of the data scale. Figure 6a shows the R values of 660 nm, 810 nm, 900 nm, 970 nm, and 1050 nm, where the blue lines represent the R_a_ calculated by the “a” color channel, and the red lines represent the R_R_ calculated by the “R” color channel. It can be seen that for the same wavelength, the variation trends of R_a_ and R_R_ are similar for 660 nm, 810 nm, and 900 nm. To further show the distribution differences of R_a_ and R_R_ for each wavelength, we calculated the variance values to analyze their stability, as shown in Figure 6b. We can observe that the variance values of parameter R_a_ at 660 nm, 810 nm, 900 nm, and 1050 nm are smaller compared to those of parameter R_R_, while the variance in R_a_ at 970 nm is larger than that in R_R_. It can be concluded that for smartphone PPG predictions of hemoglobin concentration, the “a” color channel of L*a*b color space has a greater stability than the “R” color channel of RGB color space.

### 3.2. Prediction Results of Hemoglobin Concentration

**Figure 7:**
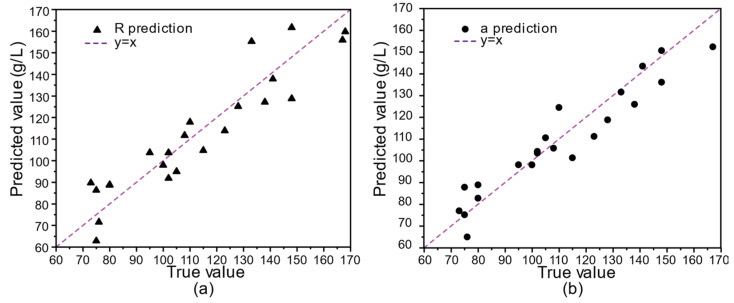
Prediction results of hemoglobin concentration using: (**a**) “R” channel and (**b**) “a” channel.

Based on the method introduced in the above section, we collected 24 groups of effective waveforms of healthy people and patients, with the hemoglobin concentration distributed between 60 mg/dL and 170 mg/dL. We counted the five wavelengths of all volunteers. Due to the amplitude modulation of the pulse wave signal caused by respiration, the R of each wavelength fluctuates periodically with the breath. To eliminate this effect, we used wavelet changes to denoise it. Retaining only the trend term R to filter out the effects of respiration, we fed the five wavelengths and true hemoglobin concentration values of 24 volunteers into a multiple linear regressor, and the predicted results are shown in Figure 7, where the horizontal axis is the true value of the hemoglobin concentration, and the longitudinal axis is the predicted value of the hemoglobin concentration. Figure 7a,b show the prediction results obtained using the “R” parameter and “a” parameter, respectively. It can be seen in Figure 7 that both the parameters can provide good hemoglobin concentration predictions for the volunteers. It should be noted that, here, the “true values” were provided by the collaborating hospital. After the smartphone measurements, a nurse took the blood samples of the volunteers for hemoglobin concentration testing.

To further compare the prediction abilities of the “R” and “a” parameters, we used *R*^2^, *RMSE* (Root Mean Square Error), and *MAPE* (Mean Absolute Percentage Error) for evaluation, as Equations (4)–(6) show,

(4) $$R^{2}=1-\frac{\sum_{i=1}^{n}{\left(y_{i}-{\hat{y}}_{i}\right)}^{2}}{\sum_{i=1}^{n}{\left(y_{i}-{\bar{y}}_{i}\right)}^{2}},\in [0,1]$$

(5) $$RMSE=\sqrt{\frac{1}{n}{\sum_{i=1}^{n}\left(y_{i}-\hat{y}\right)}^{2}},\in [0,+\infty )$$

(6) $$MAPE=\frac{100\%}{n}\sum_{i=1}^{n}\left|\frac{y_{i}-{\hat{y}}_{i}}{y_{i}}\right|,\in [0,+\infty )$$

Here, *MAPE* measures the average absolute value between the predicted and true values. The smaller the *MAPE* values of the model the better the prediction it can achieve. We compared the *R*^2^, *RMSE*, *MAPE* metrics, and Durbin–Watson test for the “a” and “R” parameters, and the results are shown in Table 1.

We can see from Table 1 that the adjusted *R*^2^ of the multivariate linear regression model obtained by using the “a” parameter of the L*a*b color space reaches 0.88, which is larger than 0.83 achieved using the “R” parameter of the RGB color space. Moreover, the *RMSE* of the multiple linear regression model obtained by using the “a” parameter is 9.04, which is smaller than the value of 10.70 obtained by using the “R” parameter. The *MAPE* obtained by using the “a” parameter is 0.068, which is much smaller than the value of 0.091 obtained by using the “R” parameter. In summary, the *R*^2^, *RMSE*, and *MAPE* of the regression results obtained by using the “a” parameter are better than those obtained by using the “R” parameter. In addition, the Durbin–Watson test was carried out to evaluate the autocorrelation problem of the independent variables. In general, the value of the Durbin–Watson test, shown in Table 1, confirms that there is no correlation between the residuals, and the subsequent improvement in the “a” parameter suggests that we should further increase the number of samples and expand the distribution range of hemoglobin concentration to further improve the prediction accuracy of the system.

## 4. Discussion

Most of the portable hemoglobin meters commercially available on the market are expensive. Although these devices can perform highly accurate hemoglobin measurements, they often require a finger prick to obtain the blood sample for testing, bringing additional infection risk for the users. In comparison to these devices, the smartphone-based biosensor proposed in this study provides a non-invasive and low-cost way of monitoring of total hemoglobin concentration. The testing results demonstrate that it has a satisfactory measurement accuracy, with irreplaceable portability and convenience. It is worth mentioning that there are other applicable methods for the direct monitoring of hemoglobin concentration using smartphone-based devices with high accuracy [36,37], whose *R*^2^ values reached 0.9810 and 0.9583, respectively. Here, our device provides a multi-wavelength optical detection method, allowing one to achieve a satisfactory measurement accuracy non-invasively. It should be pointed out that, since the signal-to-noise ratio of the smartphone camera used to respond to infrared light needs to be improved, the data collected in this study have a certain error. For the wavelength of 1300 nm where water absorption is prominent, the response of the smartphone is limited. If the signal at 1300 nm can be added, the prediction result will be more accurate. Additionally, the response speed of the smartphone camera is relatively slow. It takes 2 s to stabilize the light intensity every time the LED is switched; hence, the time division multiplexing method cannot be used in this study. The waveform signals of five wavelengths in a very short time can be obtained. This method can further eliminate the interference from human movement and the modulation induced by breathing. In addition, only 24 volunteers were involved in this study, which may have led to certain errors in the regression coefficients calculation. As mentioned above, the prediction accuracy can be improved by increasing the number of samples.

It should be noted that there are issues of various smartphones having different types of cameras and lighting bias, which may result in different light intensity and color responses under the same illumination situation. As a possible solution for future development of this method and biosensor, a color calibration scheme could be adopted. More specifically, a color and intensity calibration algorithm could be designed based on the measurement results of standard samples and light sources when different smartphones are used to make sure that the light responses of different smartphone sensors are the same. As for the issue of different camera positions in different smartphones, a variable-position light source module can be designed and used.

## 5. Conclusions

In this paper, we proposed an accurate smartphone-based biosensor for detecting human total hemoglobin concentration. The sensor utilized the camera, memory, and computing power of the smartphone itself. We collected the data of 12 normal volunteers and 12 anemia patients and developed a new multi-wavelength light source system external to the smartphone. The smartphone can provide multi-spectral signals and used a 1050 nm wavelength LED light to provide information that is closely related to the absorption of water in the blood, thereby increasing the accuracy of the regression. Through the integrated LED light, the error of motion artifacts caused by switching lights of different wavelengths was effectively reduced. We designed a smartphone-fixing bracket to reduce the position error during the measurement. The “a” parameter of the L*a* b color space was used to predict the hemoglobin concentration when processing each picture frame. The experimental results demonstrate that, compared to the “R” parameter of the RGB color space, the “a” parameter has a better performance in predicting human hemoglobin concentration using PPG signals. The results show that the smartphone-based hardware and algorithm proposed in this study can meet the accuracy requirements of non-invasive detection of hemoglobin concentration in vivo.

## Figures and Tables

**Table 1:** Comparison of the prediction results of “a” parameter and “R” parameter.

Model	Color Space	*R* ^2^	Adjusted *R*^2^	*RMSE*	*MAPE*	Durbin–Watson Test
	a (L*a*b)	0.91	0.88	9.04	0.068	1.77
	R (RGB)	0.87	0.83	10.70	0.091	2.41

## Data Availability

The data presented in this study are available on request from the corresponding author.
